# Robust Neutralizing Antibody Levels Detected after Either SARS-CoV-2 Vaccination or One Year after Infection

**DOI:** 10.3390/v13102003

**Published:** 2021-10-05

**Authors:** Stefan Glöckner, Franziska Hornung, Michael Baier, Sebastian Weis, Mathias W. Pletz, Stefanie Deinhardt-Emmer, Bettina Löffler, the CoNAN Study Group

**Affiliations:** 1 Institute of Medical Microbiology, Jena University Hospital, Friedrich-Schiller University Jena, Am Klinikum 1, 07747 Jena, Germany; franziska.hornung@med.uni-jena.de (F.H.); michael.baier@med.uni-jena.de (M.B.); stefanie.deinhardt-emmer@med.uni-jena.de (S.D.-E.); bettina.loeffler@med.uni-jena.de (B.L.); 2 Institute for Infectious Diseases and Infection Control, Jena University Hospital, Friedrich-Schiller University Jena, Am Klinikum 1, 07747 Jena, Germany; Sebastian.weis@med.uni-jena.de (S.W.); mathias.pletz@med.uni-jena.de (M.W.P.); 3 Department of Anesthesiology and Intensive Care Medicine, Jena University Hospital, Friedrich-Schiller University Jena, Am Klinikum 1, 07747 Jena, Germany; 4 Center for Sepsis Control and Care (CSCC), Jena University Hospital, Friedrich-Schiller University Jena, Am Klinikum 1, 07747 Jena, Germany; 5 Leibniz Centre for Photonics in Infection Research (LPI), 07747 Jena, Germany

**Keywords:** SARS-CoV-2, COVID-19, vaccination, neutralization, serology, antibodies, immunity

## Abstract

Humoral immunity after infection or after vaccination against severe acute respiratory syndrome coronavirus 2 (SARS-CoV-2) has been attributed a key part in mitigating the further transmission of the virus. In this study, we used a commercial anti-Spike immunoglobulin G (S-IgG) assay and developed a cell culture-based neutralization assay to understand the longitudinal course of neutralizing antibodies in both SARS-CoV2 infected or vaccinated individuals. We show that even more than one year after infection, about 78% of observed study participants remained seropositive concerning S-IgG antibodies. In addition, the serum of the individuals had stable neutralization capacity in a neutralization assay against a SARS-CoV-2 patient isolate from March 2020. We also examined volunteers after either homologous BNT162b2 prime-boost vaccination or heterologous AZD1222 prime/mRNA-based booster vaccination. Both the heterologous and the homologous vaccination regimens induced higher levels of neutralizing antibodies in healthy subjects when compared to subjects after a mild infection, showing the high effectiveness of available vaccines. In addition, we could demonstrate the reliability of S-IgG levels in predicting neutralization capacity, with 94.8% of seropositive samples showing a neutralization titer of ≥10, making it a viable yet cheap and easy-to-determine surrogate parameter for neutralization capacity.

## 1. Introduction

The ongoing pandemic of SARS-CoV-2 keeps threatening not only individual and public health but leaves its mark on almost every aspect of our lives today. As of 22 August 2021, more than 200 million confirmed cases have been reported, causing over 4 million deaths worldwide [1]. Global efforts have brought forth several vaccines with different mechanisms of action, and with over 4 billion doses administered [1], a significant part of the world’s population has developed humoral and cellular immunity against the virus. Measuring the immune response against SARS-CoV-2 both after infection and after vaccination will help guide the next necessary steps to control the pandemic.

Vaccination has proven an effective tool in the prevention of SARS-CoV-2 infections [2,3]. Both vector-based and mRNA-based vaccines approved by the European Medicines Agency generate a potent humoral and cellular immunity [4,5,6,7], inducing high levels of antibodies detectable in different assay systems.

In this study, we focused on assessing serum neutralization capacity and S-IgG antibody response longitudinally after SARS-CoV-2 infection or after vaccination. While CD4+ and CD8+ T-cells also contribute to immunity against SARS-CoV-2 [8,9], several studies demonstrated the importance of SARS-CoV-2-specific neutralizing antibodies as a protection mechanism against severe infection [10,11], with S-IgG found in almost every patient after infection. Longitudinal data of antibody concentrations for the first 6–10 months after infection exists in abundance [10,11,12,13], while evidence on the persistence of humoral immunity a year after the infection has only begun to emerge recently [14].

We used a commercial S-IgG chemiluminescence immunoassay (CLIA) and established a neutralization assay based on cell culture to demonstrate longitudinal courses of neutralizing antibody concentrations both after infection, or after vaccination to further investigate the persistence of long-term humoral immunity.

## 2. Materials and Methods

### 2.1. Study Collective

For this study, we acquired serum samples of 40 participants (m:f 21:19, median age 64, interquartile range (IQR) 53–72) infected with SARS-CoV-2 in March 2020 during one of the first outbreaks of SARS-CoV-2 in Germany in Neustadt am Rennsteig. Serum samples were initially acquired 6 weeks after a mass screening took place as part of the CoNAN study that has been described in detail in [15]. Additional follow-ups 6 months and 12 months after the initial sampling took place to enable long-term longitudinal analysis.

In addition, we recruited two groups of participants from the staff of Jena University Hospital, who received their initial vaccinations between December 2020 and February 2021. The first, homologous vaccination group (*n* = 22, m:f 6:16, median age 45, IQR 30–53) received a prime vaccination with BNT162b2 (BioNTech, Mainz, Germany) and booster vaccination with the same vaccine after 3 weeks. The second, heterologous vaccination group (*n* = 21, m:f 5:16, median age 36, IQR 32–44) received a prime vaccination with the vector-based vaccine AZD1222 (AstraZeneca, Cambridge, UK) and booster vaccination with either mRNA-1273 (Moderna, Cambridge, USA) or BNT162b2 after 12 weeks. For both vaccination groups, serial serum samples were acquired at pre-defined dates (0, 1, 2, 3, 4, 5, 8, and 16 weeks after prime vaccination). More detailed information about the study collective can be found in Supplementary Tables S1 and S2.

### 2.2. Serological Assay

Serological analyses for SARS-CoV-2 S-IgG antibodies were performed using the Liaison SARS-CoV-2 TrimericS IgG CLIA on the LiaisonXL (DiaSorin, Saluggia, Italy) following the manufacturer’s instructions. According to the manufacturer’s instruction for use, this assay detects IgG antibodies against SARS-CoV-2-specific trimeric Spike glycoprotein with an estimated sensitivity of 98.7% (153/155) at ≥15 days after the first positive RT-PCR, and an estimated specificity of 99.5% (1889/1899). Samples were defined as seropositive for determining values of ≥33.8 BAU/mL. The manufacturer states that seropositive samples showed a positive agreement of 100% (Wilson 95% CI: 97.8–100%) with a neutralization titer of ≥1:10 in a micro-neutralization assay, while the negative agreement is stated as 96.9% (Wilson 95% CI: 92.9–98.7%), making it an ideal choice for our study design.

### 2.3. Cell Culture and Virus Propagation

The SARS-CoV-2 strain SARS-CoV-2/hu/Germany/Jena-vi005588/2020 (5588) was isolated from a respiratory sample of a patient admitted to Jena University Hospital (ethics approval of the Jena University Hospital, no.: 2018–1263), propagated by using Vero 76 cells and purified by plaque assay as previously described [16]. All steps involving live viruses took place in a BSL-3 facility.

### 2.4. Neutralization Assay

The assay was performed by using Vero 76 cells seeded (0.8–1 × 10^5^ cells per well) in a 96-well plate with Eagle’s minimum essential medium (EMEM, Sigma-Aldrich, Taufkirchen, Germany) supplemented with 25 mM Hepes, 25 mM L-Glutamin and 5% fetal calve serum (FCS, Sigma-Aldrich, Taufkirchen, Germany). At first, the appropriate SARS-CoV-2 dilution yielding a distinct, microscopically visible cytopathic effect (CPE) after 48 h (hrs) was evaluated by infection of the cells with serial dilutions of the patient isolate 5588. This concentration was then chosen as our virus working dilution to be used when performing the neutralization assay with patient sera. The general workflow of the neutralization assay is shown in Supplementary Figure S1.

All sera samples were stored at −20 °C until usage and assayed after heat inactivation for 30 min (min) at 56 °C. In the next step, each serum was prediluted in medium without FCS, starting with a 1:10 dilution and further diluting in 1:1 steps until a maximum dilution of 1:1280. Afterward, each dilution was mixed with the same volume of virus working dilution and incubated for 90 min at 37 °C, 5% CO_2_. Next, the cells were washed with Dulbecco’s phosphate-buffered saline (DPBS) without calcium and magnesium (Thermo Fisher Scientific, Waltham, MA, USA), the virus-serum mixtures were added to the 96-well plate and incubated for 1 h at 37 °C, 5% CO_2_. Notably, all infection scenarios, including cell control (CC), virus-serum dilution, and virus control (VC), were performed in three replicates, resulting in two serum samples analyzed per 96-well plate. After the infection step, cells were washed once with DPBS, fresh medium was added and the cells were further incubated at 37 °C, 5% CO_2_. To analyze the effect of viral infection and incubation with the serum sample, cells were first examined using bright-field microscopy after 48 h (Supplementary Figure S2). These qualitative results with a clear detectable CPE after around 48 h post-infection in the VC were followed by the addition of WST-1 (CELL PRORO, Roche, Basel, Switzerland) for 2 h at 37 °C, 5% CO_2_. WST-1 is a stable tetrazolium salt that reacts with NADH, forming the dye formazan. Therefore, it serves as a reliable tool to quantify cell viability by measuring the resulting formazan levels at an optical density of 492 nm (reference filter 620 nm). We determined the neutralization titer by microscopy and subsequent quantitative confirmation by using the optical density values obtained of CC, VC, and serum dilutions after adding WST-1. The neutralization titer was defined as the highest dilution used in the assay at which the serum is still able to neutralize at least 50% of the CPE caused by the virus.

### 2.5. Statistical Analysis

All statistical analyses were performed using GraphPad Prism (version 8.4.3; GraphPad Software Inc., San Diego, CA, USA). IgG antibody concentrations are given as geometric means with Wilson’s 95% confidence intervals (CI) if not stated otherwise. Comparisons between two groups were performed using the Mann-Whitney U test. Significance levels in figures are represented by stars: * *p* value < 0.05; ** *p* value < 0.01; *** *p* value < 0.001, **** *p* value <0.0001. Spearman test was used to calculate the correlation coefficient of neutralization titers and S-IgG concentrations.

## 3. Results

### 3.1. Longitudinal Course of S-IgG Levels after Infection or after Vaccination

**Figure 1:**
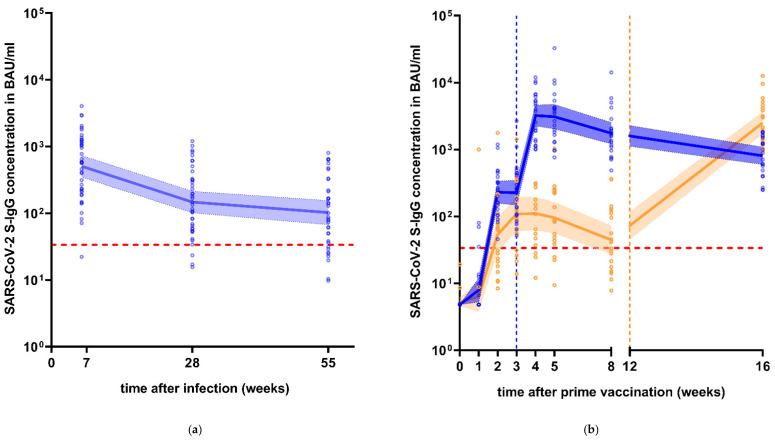
Longitudinal course of SARS-CoV-2 S-IgG antibody levels after infection and vaccination. Red dotted line = manufacturer’s positive cut-off at 33.8 BAU/mL. (**a**) S-IgG levels in 40 subjects post-infection over one year, excluding subjects at 55 weeks that received a vaccination in-between follow-ups. (**b**) S-IgG levels after homologous (blue) and heterologous (orange) vaccination. Dotted vertical lines = time of booster vaccination for homologous (blue) and heterologous (orange) vaccination regimen.

About seven weeks after infection, 39/40 (97.5%) of infected subjects tested positive for SARS-CoV-2 S-IgG antibodies. While S-IgG levels were at 507.7 BAU/mL (95% CI: 349.5–737.4) at this point, the concentrations fell distinctly to 147.6 BAU/mL (95% CI: 101.9–213.8, *p* < 0.0001) at the 6-months follow-up and 102.5 BAU/mL (95% CI: 67.6–155.5, *p* = 0.2066) at the 12-months follow-up, respectively (Figure 1a). At the 6-months follow-up, two out of 39 formerly positive subjects tested negative, an additional five subjects became seronegative at the 12-months follow-up. Excluding four subjects who received a vaccination between first and second follow-up, a total of 77.8% (28/36) of subjects were seropositive concerning S-IgG 55 weeks after infection.

All participants of the vaccination groups developed S-IgG antibody levels at least ten times above the manufacturer’s positive cut-off after administration of the booster vaccine (Figure 1b). While all serum samples showed positive signals within three weeks after prime vaccination with BNT162b2, 14.3% (3/21) of subjects initially vaccinated with AZD1222 remained seronegative until the booster vaccination.

**Figure 2:**
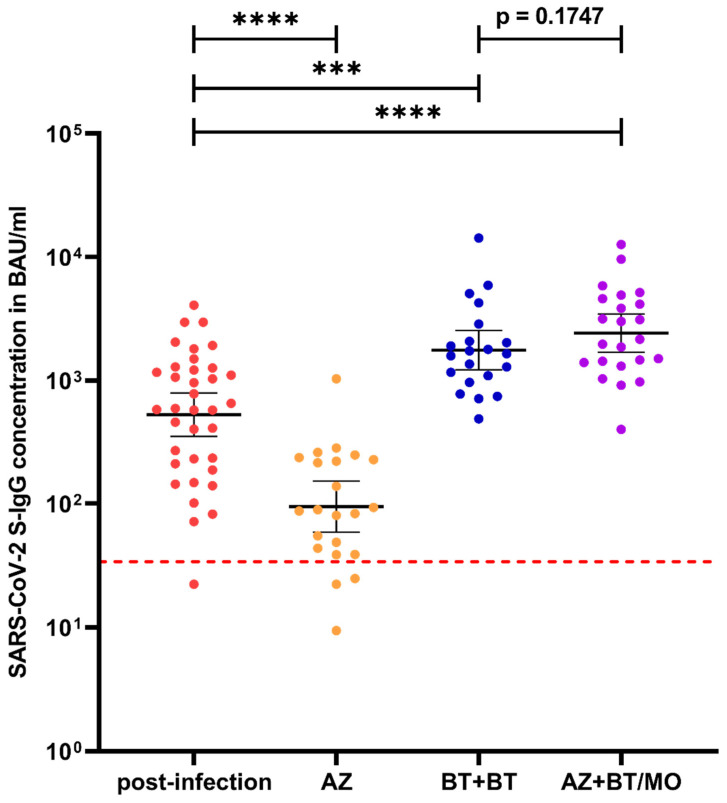
Comparison of S-IgG antibody concentrations about seven weeks post-infection (red), five weeks after prime vaccination with AZD1222 (orange) or BNT162b2 (=two weeks after homologous booster vaccination, blue), and four weeks after heterologous booster vaccination (purple). Red dotted line = manufacturer’s positive cut-off at 33.8 BAU/mL. Abbreviations: AZ = AZD1222, BT = BNT162b2, MO = mRNA-1273. Significance levels: *** *p* value < 0.001, **** *p* value < 0.0001.

Homologous and heterologous vaccination regimen induced S-IgG antibody levels of 1755 BAU/mL (95% CI: 1219–2527) and 2411 BAU/mL (95% CI: 1689–3441), respectively, 4–5 weeks after booster vaccination. While the heterologous regimen showed no significant difference in outcome when compared to the homologous regimen (*p* = 0.1747), both vaccination regimens induced significantly higher (*p* = 0.0002) S-IgG concentrations than found in the convalescent group (Figure 2). At the final follow-up, 13 weeks after booster vaccination, S-IgG levels decreased to 806.6 (95% CI: 598–1087, *p* = 0.026) in the homologous vaccination group but remained distinctly above the initial levels found in convalescent sera about seven weeks after infection.

### 3.2. Neutralization Capacity after Infection and Vaccination

**Figure 3:**
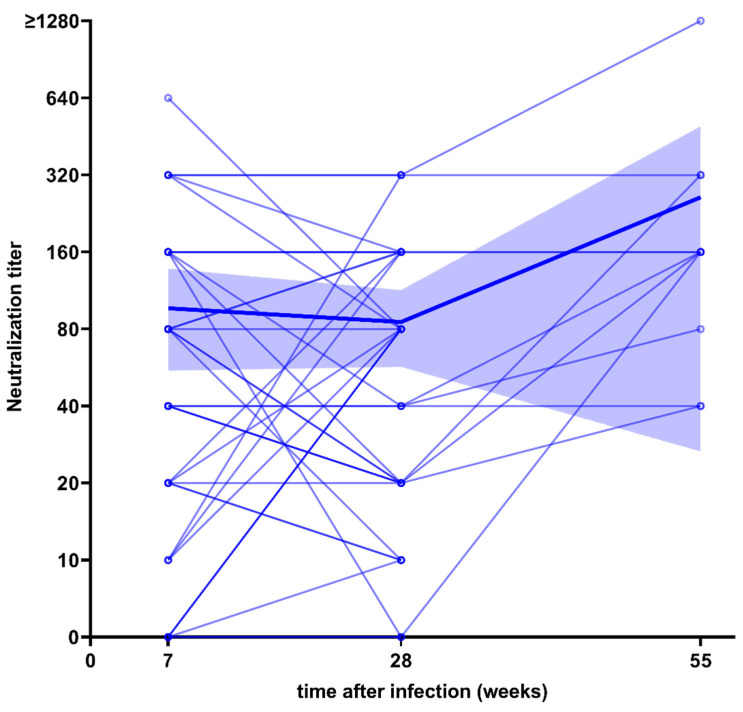
Longitudinal course of neutralization titers in 40 serum samples of participants after infection with SARS-CoV-2 over 28 weeks, with data for 11 serum samples over 55 weeks. The highlighted blue graph shows the mean neutralization titer with its 95% confidence interval.

All 40 serum samples of infected subjects about seven weeks after infection were also tested for their neutralization capacity with the aforementioned neutralization assay. We found that 85% (36/40) of the subjects had measurable neutralization activity with an average neutralization titer of 96.5 (95% CI: 54.9–138.1), remaining stable at the 6-months follow-up with 85.3 (95% CI: 56.8–113.7, *p* = 0.8050). Further 11 serum samples taken at the 12-months follow-up, representing the full range of S-IgG concentrations measured at that point, still showed measurable neutralization capacity in all tested samples (Figure 3).

**Figure 4:**
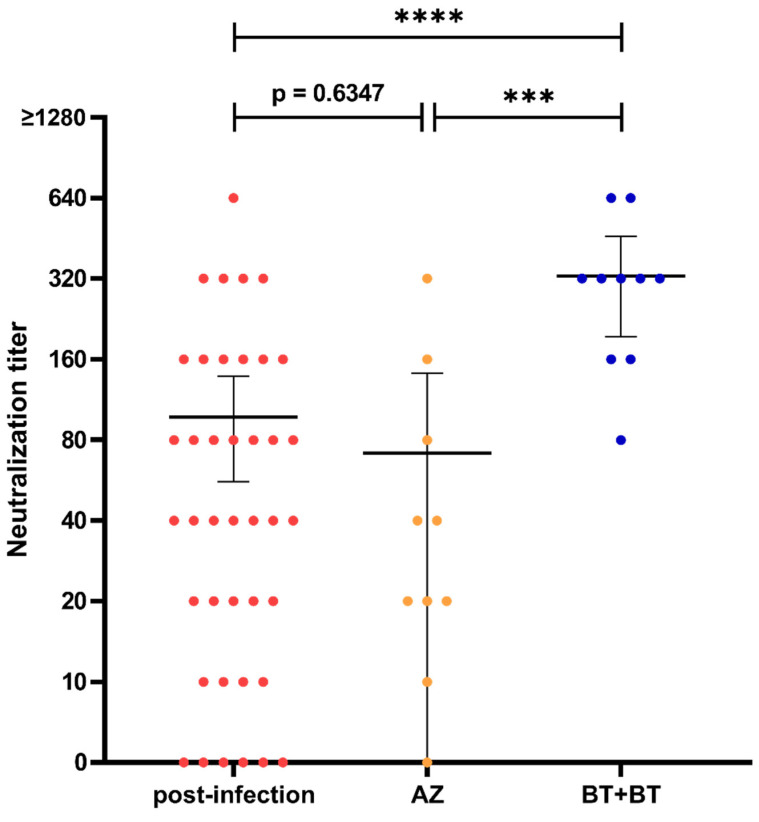
Comparison of neutralization titers of serum samples seven weeks post-infection (red) and five weeks after prime vaccination with AZD1222 (orange) or BNT162b2 (= two weeks after homologous booster vaccination, blue). Abbreviations: AZ = AZD1222, BT = BNT162b2, MO = mRNA-1273. Significance levels: *** *p* value < 0.001, **** *p* value < 0.0001.

Furthermore, we determined the efficacy of both prime and booster vaccination concerning the induction of neutralizing antibodies and selected 10 representative serum samples based on their S-IgG levels after prime and booster vaccination, respectively. Prime vaccination with AZD1222 induced neutralizing capacity after 5 weeks in levels comparable to those after infection (mean neutralization titer 71.0 vs. 96.5, Mann-Whitney U test *p* = 0.6347, Figure 4). Homologous prime-boost vaccination generated distinctly higher mean neutralization titers of 328.0 (95% CI: 194.6–461.4) 2 weeks after booster vaccination, with every selected sample reaching a neutralizing titer of at least 80.

We also observed the effect of booster vaccination administered between the two follow-ups to four convalesced study participants. In this small subgroup, both mean S-IgG concentrations and mean neutralization capacity were higher by at least one magnitude at the 12-months follow-up when compared to the 6-months follow-up, showing a distinct effect of the applied vaccine on humoral response in convalesced subjects (Supplementary Table S3).

### 3.3. Correlation between S-IgG Levels and Neutralization Capacity

**Figure 5:**
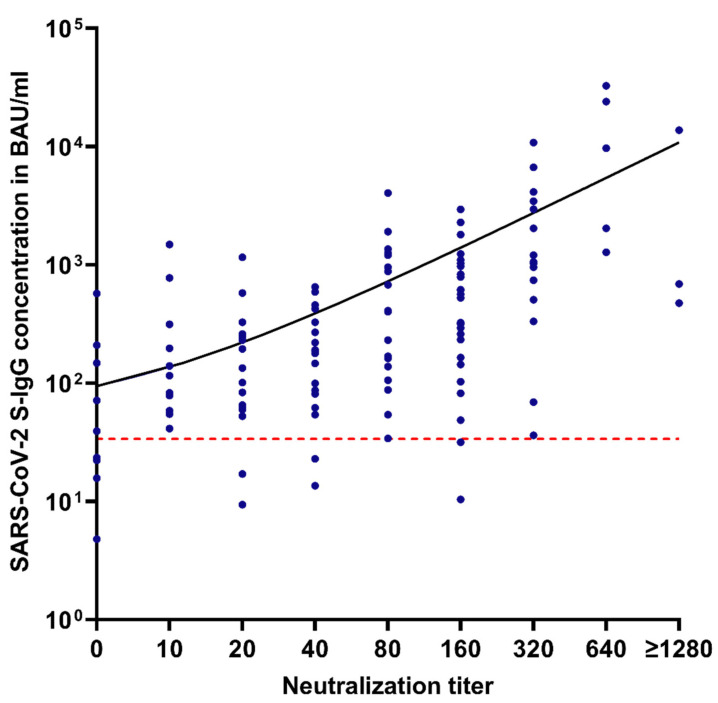
Correlation of neutralization titers and SARS-CoV-2 S-IgG antibody concentrations in BAU/mL. Red dotted line = manufacturer’s positive cut-off at 33.8 BAU/mL.

S-IgG antibody levels measured with the SARS-CoV-2 TrimericS IgG CLIA showed correlation with neutralization capacity (Spearman r = 0.67, *p* < 0.0001, Figure 5). The correlation was even higher in samples acquired within about 7 weeks after infection (r = 0.73, *p* < 0.0001), but continuously decreased in the 6 and 12-months follow-ups (r = 0.65, *p* < 0.0001 and r = 0.58, *p* = 0.0263, respectively).

Seropositive samples, defined by an S-IgG concentration of ≥ 33.8 BAU/mL, showed a positive result in our neutralization assay with a titer of ≥10 in 94.8% (110/116) of tested samples.

## 4. Discussion

In this study, we assessed longitudinally the humoral immune response in individuals after infection with or vaccination against SARS-CoV-2. Our data show that S-IgG is found in levels distinctly higher than the positive threshold in almost all participants about seven weeks after infection, followed by a steady decline over months consistent with studies released over the past year [17,18,19]. Even after more than one year, the majority of convalesced patients remained S-IgG seropositive, indicating a more stable and long-lasting antibody response than evidence from the earlier stages of the pandemic might have suggested [20,21].

Reports on the longitudinal course of neutralizing antibody levels have been inconsistent, describing both losses of neutralizing capacity over time in serum samples collected from convalesced COVID-19 patients [12,22] and relative stability over months [9,23]. Many of the studies examining the neutralization capacity of sera against SARS-CoV-2 used pseudovirus particles as a surrogate to determine neutralization titers, thus avoiding the necessity of using a BSL-3 facility [10,24,25,26]. While this approach reduces expenses and allows for higher throughput, our assay has the benefit of showing the interaction of replicating the virus with viable cells and serum components during infection. Taking into consideration that not all mechanisms involved in neutralization are necessarily covered by pseudoviruses, using a SARS-CoV-2 strain isolated from a patient specimen might therefore provide data that reflect the in vivo situation more closely. The results of our neutralization assay confirm the persistence of neutralization capacity in convalescent serum samples over 55 weeks.

Due to the limited applicability of our time- and resource-consuming neutralization assay for everyday use in a clinical setting, we investigated the correlation between S-IgG levels and neutralization titers. Several commercially available IgG assays, especially those available early in the pandemic, showed poor correlation with neutralization assays [27,28]. While Spike glycoprotein remains the most important target for neutralizing antibodies, not every S-IgG antibody possesses the ability to inhibit the Spike glycoprotein from binding to ACE2 and therefore stop cell entry. The S-IgG CLIA performed in this study used the approach of targeting trimeric Spike glycoprotein. Targeting specific domains involved in neutralization, like the RBD or the N-terminal domain [29,30], might allow an even better prediction of neutralization capacity. Still, S-IgG levels measured in our study proved as an easy-to-determine, viable surrogate parameter for neutralization capacity both after vaccination and infection. Yet, even with standardized commercial assays, clinical interpretation of measured neutralizing antibody levels or their correlates remains difficult due to the lack of reliable, evidence-based thresholds indicating protection from severe disease. While predictive models do exist [31], further observational studies with large numbers of participants and a high frequency of sampling are needed.

In addition to immunity through infection, vaccination against SARS-CoV-2 plays a vital role in achieving herd immunity and individual protection from severe disease. During the past year, several vaccines with different mechanisms of action have been used excessively to restrict the further spreading of SARS-CoV-2. To achieve effective and lasting immunity, vaccines should induce both a cellular and a humoral immune response at least comparable to the response caused by the pathogen they are targeted at. We identified distinctly higher levels of neutralizing antibodies in subjects after administration of the recommended prime-boost vaccination regimen than after prime vaccination or infection, indicating stronger protection against infection and severe disease. Due to the generally mild course of disease in our convalescent group and slight differences between the time of sampling when compared to the vaccinated groups, the effect of the booster vaccination in comparison to infection might be overestimated in our study. While our data show non-inferiority of heterologous vaccination when compared to homologous prime-boost vaccination, the use of BNT162b2 in the homologous group and the predominant use of mRNA-1273 for heterologous vaccination might be problematic, especially in the light of a recent study showing higher immunogenicity of the mRNA-1273 vaccination when compared to BNT162b2 [32]. In our study, this effect might be altered due to the difference in time of application of the booster vaccination and due to different vaccines used for prime vaccination. Earlier data had already shown similar results to those presented here when comparing an AZD1222/BNT162b2 combination with a homologous BNT162b2 prime-boost vaccination [33].

Recruiting representative vaccination cohorts in a healthcare system with women making up about 80% of the workers proved to be difficult, and the resulting dysbalance between male and female participant numbers in the vaccination cohorts may seem problematic at first, but earlier studies have shown no distinct difference in the humoral response to SARS-CoV-2 vaccination and infection between sexes in adult patients of working age [34,35].

Vaccine-induced immune response has been shown to be robust and stable over several months [36], with mRNA-based vaccines being effective at inducing persistent and robust germinal centers binding Spike glycoprotein [37]. With regard to emerging SARS-CoV-2 strains with modified Spike structure, lowering effectivity of neutralizing antibodies induced by vaccination or infection with other SARS-CoV-2 variants [38,39,40], a recommendation for convalesced COVID-19 patients to receive a booster vaccination, increasing neutralizing antibody levels and thus lowering the risk of reinfection and severe disease, should be considered especially for vulnerable groups. While we can show a decline of S-IgG antibody levels over time in vaccinated subjects analogously to the antibody course in convalesced subjects, loss of circulating IgG does not necessarily mean the disappearance of protection against severe infection. A recent study has shown gradually increasing RBD- and stable Spike IgG+ memory B-cell (MBC) levels in COVID-19 patients up to 250 days after symptom onset [41], possibly inducing a quick increase in neutralizing S-IgG antibody levels after breakthrough infection. This might decrease the risk of severe infection even in patients with initially low levels of circulating neutralizing antibodies and needs to be considered when planning future immunization programs.

In conclusion, we showed stable neutralization capacity in convalescent serum samples over 55 weeks, correlating well with S-IgG antibody concentrations. In addition, we demonstrated the high efficacy of homologous and heterologous prime-boost vaccination regimens at inducing S-IgG antibodies, offering an optimistic perspective on the outcome of global vaccination programs. Clear thresholds of neutralizing antibody levels indicating immunity from infection or severe disease remain to be determined in further research to answer the important question of the necessity of future booster vaccinations.

## Data Availability

The data presented in this study are available in Supplementary Tables S1–S4.

## Supplementary Materials

The following are available online at https://www.mdpi.com/article/10.3390/v13102003/s1, Figure S1: Workflow of the neutralization assay, Figure S2: Determination of the neutralization titer via bright-field microscopy, Table S1: Basic data of recruited subjects after SARS-CoV-2 infection, Table S2: Basic data of recruited subjects after SARS-CoV-2 vaccination, Table S3: Antibody data after infection, Table S4: Antibody data after vaccination.
