# Supplementary material for: Robust Neutralizing Antibody Levels Detected after Either SARS-CoV-2 Vaccination or One Year after Infection
Source: Viruses. 2021 Oct 5;13(10):2003. doi: 10.3390/v13102003 (PMC8537517; doi:10.3390/v13102003)
Supplement: Supplementary file 1 [file viruses-13-02003-s001.zip › SupplementaryTables.pdf]

Supplementary Table S1. Basic data of recruited subjects after SARS-CoV-2 infection.

ID	sex	age	severity of disease	vaccinated after 6-months follow-up
POSTINF-1	f	71	asymptomatic	no
POSTINF-2	m	64	asymptomatic	no
POSTINF-3	m	45	asymptomatic	no
POSTINF-4	f	52	asymptomatic	no
POSTINF-5	m	73	asymptomatic	no
POSTINF-6	m	56	asymptomatic	no
POSTINF-7	f	62	mild	no
POSTINF-8	m	67	mild	no
POSTINF-9	f	51	mild	no
POSTINF-10	m	68	mild	no
POSTINF-11	f	63	mild	no
POSTINF-12	f	39	mild	no
POSTINF-13	f	68	mild	no
POSTINF-14	m	53	mild	no
POSTINF-15	m	58	mild	no
POSTINF-16	f	83	mild	no
POSTINF-17	m	78	mild	no
POSTINF-18	m	76	mild	no
POSTINF-19	f	58	mild	no
POSTINF-20	f	54	mild	no
POSTINF-21	f	52	mild	no
POSTINF-22	m	59	mild	no
POSTINF-23	m	50	mild	no
POSTINF-24	m	58	mild	no
POSTINF-25	m	69	mild	no
POSTINF-26	f	81	mild	no
POSTINF-27	m	72	moderate	no
POSTINF-28	f	45	moderate	no
POSTINF-29	m	68	moderate	no
POSTINF-30	m	41	moderate	no
POSTINF-31	f	68	moderate	no
POSTINF-32	f	71	moderate	no
POSTINF-33	m	74	moderate	no
POSTINF-34	m	80	moderate	no
POSTINF-35	f	76	moderate	no
POSTINF-36	m	62	moderate	no
POSTINF-37	f	53	mild	*yes*
POSTINF-38	f	55	mild	*yes*
POSTINF-39	f	70	mild	*yes*
POSTINF-40	m	73	mild	*yes*

S**upplementary Table S2**. Basic data of recruited subjects after SARS-CoV-2 vaccination. AZ = AZD1222, BT = BNT162b2, MO = mRNA-1273.

ID	sex	age	vaccination regimen	days between prime and booster vaccination
Homologous vaccination				
HOMVAC-1	m	27	BT/BT	21
HOMVAC-2	f	62	BT/BT	21
HOMVAC-3	m	35	BT/BT	21
HOMVAC-4	f	45	BT/BT	21
HOMVAC-5	f	26	BT/BT	21
HOMVAC-6	f	43	BT/BT	21
HOMVAC-7	f	44	BT/BT	21
HOMVAC-8	f	30	BT/BT	21
HOMVAC-9	f	62	BT/BT	21
HOMVAC-10	f	36	BT/BT	21
HOMVAC-11	f	60	BT/BT	21
HOMVAC-12	f	48	BT/BT	21
HOMVAC-13	f	49	BT/BT	21
HOMVAC-14	f	53	BT/BT	21
HOMVAC-15	f	45	BT/BT	21
HOMVAC-16	m	29	BT/BT	21
HOMVAC-17	m	26	BT/BT	21
HOMVAC-18	f	46	BT/BT	21
HOMVAC-19	m	28	BT/BT	21
HOMVAC-20	f	40	BT/BT	21
HOMVAC-21	m	53	BT/BT	19
HOMVAC-22	f	59	BT/BT	21
Heterologous vaccination				
HETVAC-1	f	36	AZ/BT	85
HETVAC-2	m	30	AZ/MO	84
HETVAC-3	f	41	AZ/MO	84
HETVAC-4	f	34	AZ/BT	52
HETVAC-5	f	53	AZ/MO	84
HETVAC-6	f	56	AZ/MO	84
HETVAC-7	m	32	AZ/MO	84
HETVAC-8	f	38	AZ/MO	84
HETVAC-9	f	28	AZ/MO	84
HETVAC-10	f	47	AZ/MO	84
HETVAC-11	f	26	AZ/MO	84
HETVAC-12	f	35	AZ/MO	84
HETVAC-13	m	25	AZ/MO	84
HETVAC-14	f	34	AZ/MO	84
HETVAC-15	f	41	AZ/MO	84
HETVAC-16	f	36	AZ/MO	84
HETVAC-17	f	31	AZ/MO	84
HETVAC-18	m	40	AZ/MO	84
HETVAC-19	f	46	AZ/MO	84
HETVAC-20	m	36	AZ/MO	82
HETVAC-21	f	57	AZ/MO	84

Supplementary Table S3. Longitudinal course of S-IgG concentrations and neutralization capacity of 40 subjects after SARS-CoV-2 infection.

ID	7 weeks post-infection		6-months follow-up		12-months follow-up	
	S-IgG in BAU/ml	neutralization titer	S-IgG in BAU/ml	neutralization titer	S-IgG in BAU/ml	neutralization titer
POSTINF-1	591	40	134	20	101	N/A
POSTINF-2	1060	320	106	80	49.7	N/A
POSTINF-3	210	0	39.3	0	29.1	N/A
POSTINF-4	148	0	54.2	80	50.4	N/A
POSTINF-5	579	20	195	20	165	160
POSTINF-6	144	160	52.5	20	62	40
POSTINF-7	1800	160	179	40	87.6	80
POSTINF-8	573	0	169	80	167	N/A
POSTINF-9	1910	80	328	20	221	N/A
POSTINF-10	231	20	34.1	80	25.9	N/A
POSTINF-11	1210	320	508	320	476	1280
POSTINF-12	71.5	0	197	10	168	N/A
POSTINF-13	573	0	107	80	144	N/A
POSTINF-14	402	80	58.5	10	38.4	N/A
POSTINF-15	777	10	315	160	140	N/A
POSTINF-16	1030	160	293	160	213	N/A
POSTINF-17	1280	640	1210	80	651	N/A
POSTINF-18	140	10	69.2	320	82.1	N/A
POSTINF-19	101	20	41.3	10	24.9	N/A
POSTINF-20	234	20	116	10	78.1	N/A
POSTINF-21	651	40	147	40	31.6	160
POSTINF-22	269	40	54.2	40	22.9	40
POSTINF-23	1260	80	880	80	803	N/A
POSTINF-24	22.3	0	23.5	0	19.8	N/A
POSTINF-25	958	80	103	160	61.1	N/A
POSTINF-26	410	80	65.4	20	36.3	320
POSTINF-27	2950	160	833	160	638	N/A
POSTINF-28	82	160	15.7	0	10.4	160
POSTINF-29	2960	320	741	320	333	320
POSTINF-30	458	40	83.4	20	73.6	N/A
POSTINF-31	2040	320	613	160	639	N/A
POSTINF-32	1160	20	260	160	325	160
POSTINF-33	1490	10	231	80	130	N/A
POSTINF-34	1100	160	1030	160	458	N/A
POSTINF-35	4060	80	617	160	564	160
POSTINF-36	187	40	17.1	20	9.76	N/A
POSTINF-37	191	40	426	40	1270	80
POSTINF-38	314	10	82.7	10	24000	640
POSTINF-39	1360	80	317	160	13800	1280
POSTINF-40	220	40	59.7	20	2040	640

Supplementary Table S4. S-IgG concentrations of 43 subjects 0-16 weeks after vaccination.

ID	S-IgG concentration in BAU/ml over time (in weeks)							
	0	1	2	3	4	5	8	16
Homologous vaccination								
HOMVAC-1	4.96	80.1	1060	1110	6640	4140	2850	1830
HOMVAC-2	<4.81	<4.81	330	408	9870	10800	5040	1640
HOMVAC-3	<4.81	<4.81	89.8	64.8	1140	957	963	610
HOMVAC-4	<4.81	69.7	163	108	1400	1280	1090	705
HOMVAC-5	<4.81	<4.81	476	437	10500	9690	5880	1590
HOMVAC-6	<4.81	6.66	N/A	261	3990	N/A	2020	1220
HOMVAC-7	<4.81	<4.81	82.1	85	2130	2670	1780	397
HOMVAC-8	<4.81	12.5	1190	2660	12000	32700	14200	1770
HOMVAC-9	<4.81	N/A	45.8	51.9	1120	974	774	402
HOMVAC-10	<4.81	13.6	248	259	1910	1820	1280	489
HOMVAC-11	<4.81	9.84	159	106	1910	1650	742	250
HOMVAC-12	<4.81	<4.81	484	539	4930	3450	1580	891
HOMVAC-13	<4.81	<4.81	128	182	5310	4280	1640	1000
HOMVAC-14	<4.81	<4.81	200	103	1320	1310	710	249
HOMVAC-15	<4.81	<4.81	103	76.8	1000	762	486	270
HOMVAC-16	<4.81	35.1	263	228	6130	3980	2070	1230
HOMVAC-17	<4.81	<4.81	446	492	6720	6690	4240	1810
HOMVAC-18	<4.81	<4.81	391	446	4570	5360	N/A	1510
HOMVAC-19	<4.81	<4.81	317	298	2550	2290	1350	568
HOMVAC-20	N/A	<4.81	321	244	5350	3680	1730	1230
HOMVAC-21	N/A	<4.81	105	139	3750	4210	1900	1190
HOMVAC-22	<4.81	<4.81	148	136	1020	1970	1160	602
Heterologous vaccination								
HETVAC-1	<4.81	<4.81	66.8	122	150	248	357	4590
HETVAC-2	<4.81	<4.81	30.7	391	309	236	56.2	12600
HETVAC-3	<4.81	<4.81	66.3	193	177	N/A	111	3840
HETVAC-4	<4.81	<4.81	112	344	304	227	142	N/A
HETVAC-5	<4.81	<4.81	10.6	13.8	12.1	9.4	7.82	1500
HETVAC-6	8.59	8.81	68.1	170	N/A	138	95.5	1860
HETVAC-7	<4.81	<4.81	91.9	N/A	130	93.2	42.2	3000
HETVAC-8	<4.81	<4.81	8.41	26.2	37.1	43.4	41.5	2150
HETVAC-9	<4.81	<4.81	23.4	54	46.9	38.5	17.5	399
HETVAC-10	<4.81	<4.81	112	130	109	82.8	15.2	3150
HETVAC-11	<4.81	<4.81	21.1	83.6	N/A	86.9	30.5	5840
HETVAC-12	<4.81	<4.81	14.8	93.9	108	89.2	11.4	1030
HETVAC-13	19.2	1000	1770	1420	1140	1030	363	4150
HETVAC-14	<4.81	<4.81	215	N/A	273	221	45.2	3100
HETVAC-15	<4.81	<4.81	63.2	63.5	55.2	48.6	27.9	969
HETVAC-16	<4.81	<4.81	11	31.2	28.1	24.7	14.9	1960
HETVAC-17	<4.81	<4.81	152	381	313	260	129	9590
HETVAC-18	<4.81	<4.81	77.2	N/A	N/A	N/A	33.1	1460
HETVAC-19	<4.81	<4.81	28	56.6	64.3	54.7	21.4	1300
HETVAC-20	<4.81	<4.81	18.1	21.4	24.6	22.3	14.2	1390
HETVAC-21	<4.81	<4.81	206	336	313	283	123	5140
