# Supplementary material for: Robust Neutralizing Antibody Levels Detected after Either SARS-CoV-2 Vaccination or One Year after Infection
Source: Viruses. 2021 Oct 5;13(10):2003. doi: 10.3390/v13102003 (PMC8537517; doi:10.3390/v13102003)
Supplement: Supplementary file 1 [file viruses-13-02003-s001.zip › supplements.pdf]

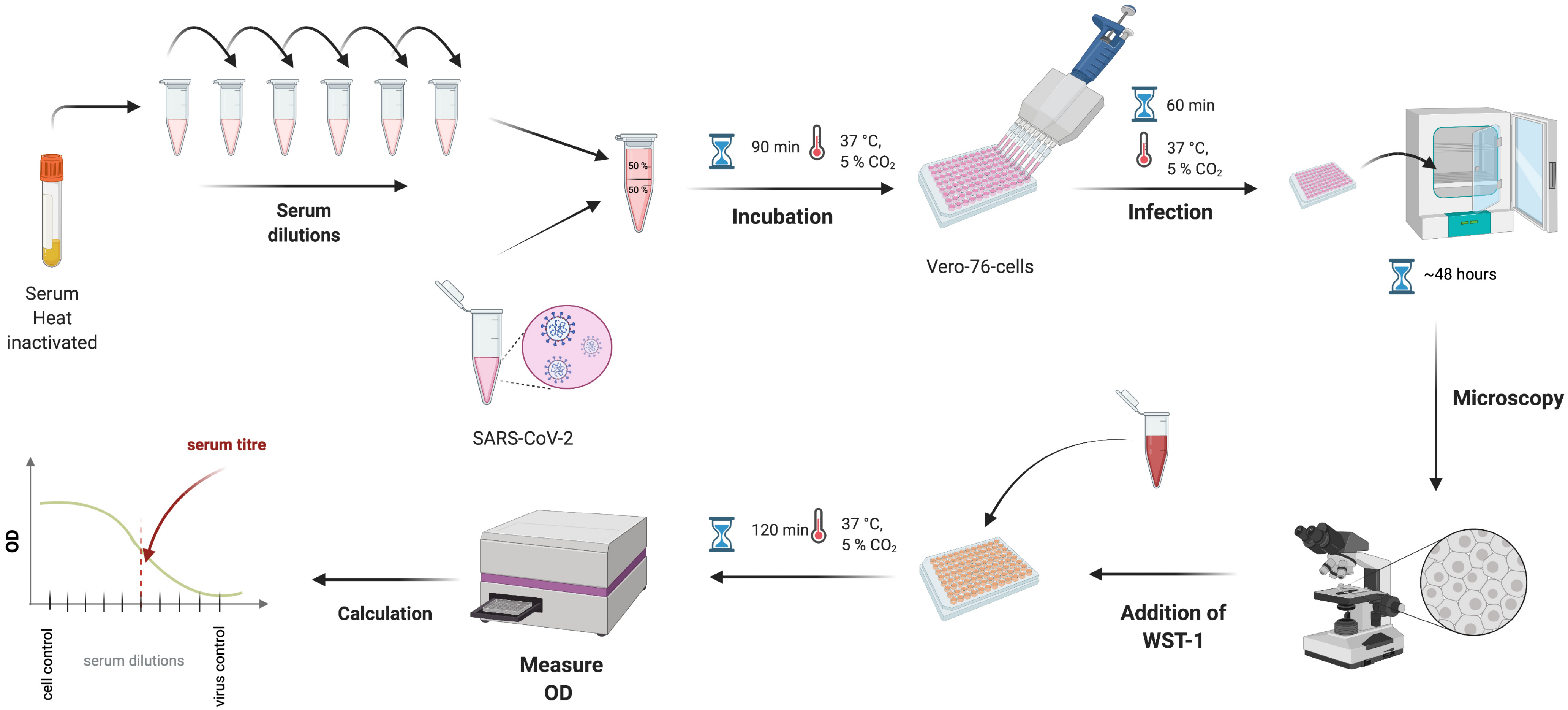

Supplementary Figure S1. Workflow of the neutralization assay. Figure created with BioRender.com

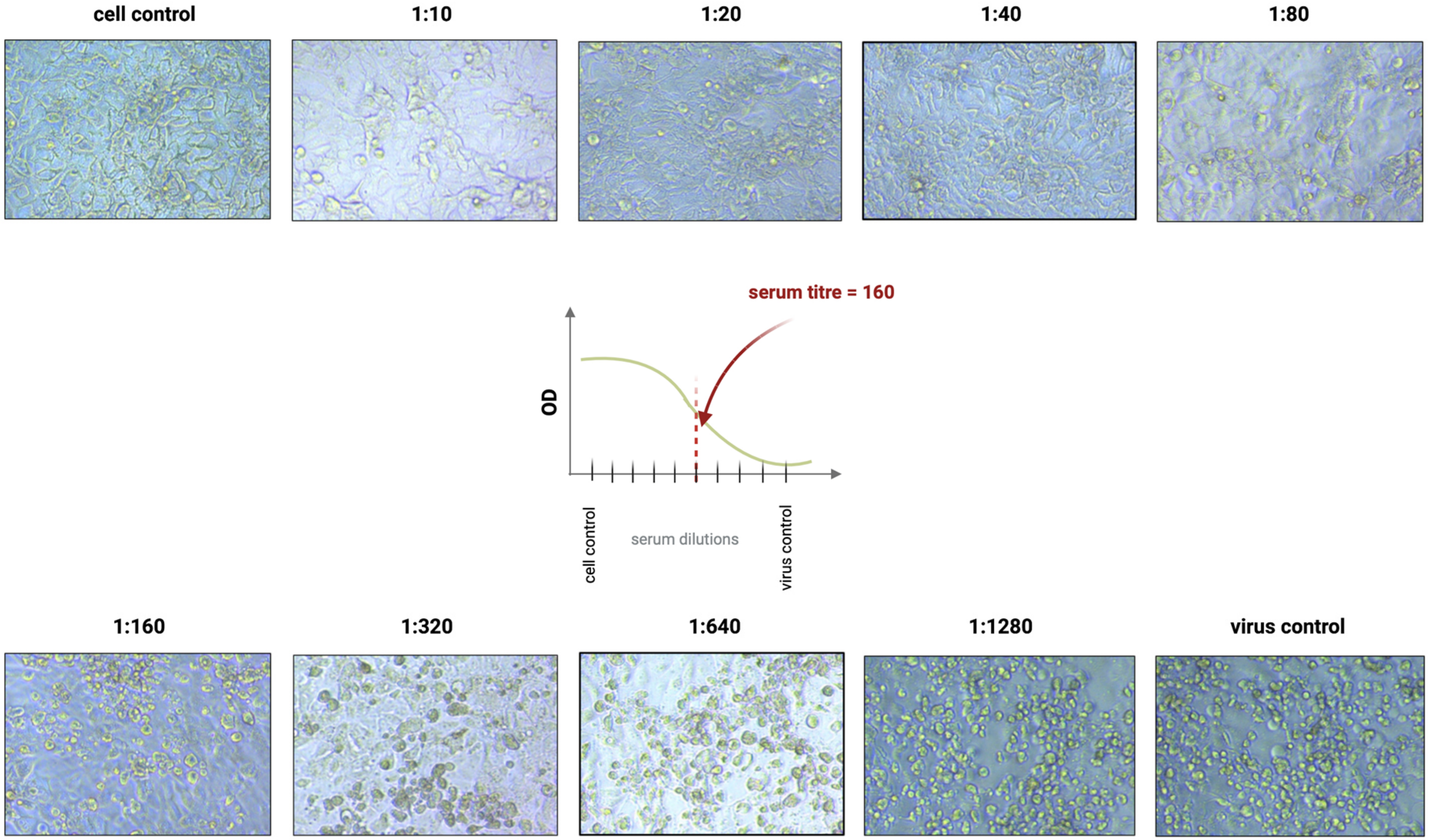

Supplementary Figure S2. Bright-field microscopical images of Vero 76 cells after 48 h of infection, prior to the WST-1-assay. These images reflect the qualitative outcome of viable and intact cells in the cell control (CC), the sufficient cytopathic effect of the virus control (VC) without serum and particularly the effect of the diluted patient serum on the cell viability. For this specific patient, the cells incubated with virus and 1:160 serum dilution show first cytopathic signs. This microscopical outcome was subsequently validated with quantitative OD values gained by the WST-assay. The calculated neutralization titer, determined as the serum dilution at which cell viability was reduced by 50 % corresponding to the values for the CC and the VC, confirmed the titer of 160. Figure created with BioRender.com

Supplementary Table S1. Basic data of recruited subjects after SARS-CoV-2 infection.

ID	sex	age	severity of disease	vaccinated after 6-months follow-up
POSTINF-1	f	71	asymptomatic	no
POSTINF-2	m	64	asymptomatic	no
POSTINF-3	m	45	asymptomatic	no
POSTINF-4	f	52	asymptomatic	no
POSTINF-5	m	73	asymptomatic	no
POSTINF-6	m	56	asymptomatic	no
POSTINF-7	f	62	mild	no
POSTINF-8	m	67	mild	no
POSTINF-9	f	51	mild	no
POSTINF-10	m	68	mild	no
POSTINF-11	f	63	mild	no
POSTINF-12	f	39	mild	no
POSTINF-13	f	68	mild	no
POSTINF-14	m	53	mild	no
POSTINF-15	m	58	mild	no
POSTINF-16	f	83	mild	no
POSTINF-17	m	78	mild	no
POSTINF-18	m	76	mild	no
POSTINF-19	f	58	mild	no
POSTINF-20	f	54	mild	no
POSTINF-21	f	52	mild	no
POSTINF-22	m	59	mild	no
POSTINF-23	m	50	mild	no
POSTINF-24	m	58	mild	no
POSTINF-25	m	69	mild	no
POSTINF-26	f	81	mild	no
POSTINF-27	m	72	moderate	no
POSTINF-28	f	45	moderate	no
POSTINF-29	m	68	moderate	no
POSTINF-30	m	41	moderate	no
POSTINF-31	f	68	moderate	no
POSTINF-32	f	71	moderate	no
POSTINF-33	m	74	moderate	no
POSTINF-34	m	80	moderate	no
POSTINF-35	f	76	moderate	no
POSTINF-36	m	62	moderate	no
POSTINF-37	f	53	mild	***yes***
POSTINF-38	f	55	mild	***yes***
POSTINF-39	f	70	mild	***yes***
POSTINF-40	m	73	mild	***yes***

S**upplementary Table S2**. Basic data of recruited subjects after SARS-CoV-2 vaccination. AZ = AZD1222, BT = BNT162b2, MO = mRNA-1273.

**ID**	**sex**	**age**	**vaccination regimen**	**days between prime and booster vaccination**
Homologous vaccination				
HOMVAC-1	m	27	BT/BT	21
HOMVAC-2	f	62	BT/BT	21
HOMVAC-3	m	35	BT/BT	21
HOMVAC-4	f	45	BT/BT	21
HOMVAC-5	f	26	BT/BT	21
HOMVAC-6	f	43	BT/BT	21
HOMVAC-7	f	44	BT/BT	21
HOMVAC-8	f	30	BT/BT	21
HOMVAC-9	f	62	BT/BT	21
HOMVAC-10	f	36	BT/BT	21
HOMVAC-11	f	60	BT/BT	21
HOMVAC-12	f	48	BT/BT	21
HOMVAC-13	f	49	BT/BT	21
HOMVAC-14	f	53	BT/BT	21
HOMVAC-15	f	45	BT/BT	21
HOMVAC-16	m	29	BT/BT	21
HOMVAC-17	m	26	BT/BT	21
HOMVAC-18	f	46	BT/BT	21
HOMVAC-19	m	28	BT/BT	21
HOMVAC-20	f	40	BT/BT	21
HOMVAC-21	m	53	BT/BT	19
HOMVAC-22	f	59	BT/BT	21
Heterologous vaccination				
HETVAC-1	f	36	AZ/BT	85
HETVAC-2	m	30	AZ/MO	84
HETVAC-3	f	41	AZ/MO	84
HETVAC-4	f	34	AZ/BT	52
HETVAC-5	f	53	AZ/MO	84
HETVAC-6	f	56	AZ/MO	84
HETVAC-7	m	32	AZ/MO	84
HETVAC-8	f	38	AZ/MO	84
HETVAC-9	f	28	AZ/MO	84
HETVAC-10	f	47	AZ/MO	84
HETVAC-11	f	26	AZ/MO	84
HETVAC-12	f	35	AZ/MO	84
HETVAC-13	m	25	AZ/MO	84
HETVAC-14	f	34	AZ/MO	84
HETVAC-15	f	41	AZ/MO	84
HETVAC-16	f	36	AZ/MO	84
HETVAC-17	f	31	AZ/MO	84
HETVAC-18	m	40	AZ/MO	84
HETVAC-19	f	46	AZ/MO	84
HETVAC-20	m	36	AZ/MO	82
HETVAC-21	f	57	AZ/MO	84

Supplementary Table S3. Longitudinal course of S-IgG concentrations and neutralization capacity of 40 subjects after SARS-CoV-2 infection.

ID	7 weeks post-infection		6-months follow-up		12-months follow-up	
	S-IgG in BAU/ml	neutralization titer	S-IgG in BAU/ml	neutralization titer	S-IgG in BAU/ml	neutralization titer
POSTINF-1	591	40	134	20	101	N/A
POSTINF-2	1060	320	106	80	49.7	N/A
POSTINF-3	210	0	39.3	0	29.1	N/A
POSTINF-4	148	0	54.2	80	50.4	N/A
POSTINF-5	579	20	195	20	165	160
POSTINF-6	144	160	52.5	20	62	40
POSTINF-7	1800	160	179	40	87.6	80
POSTINF-8	573	0	169	80	167	N/A
POSTINF-9	1910	80	328	20	221	N/A
POSTINF-10	231	20	34.1	80	25.9	N/A
POSTINF-11	1210	320	508	320	476	1280
POSTINF-12	71.5	0	197	10	168	N/A
POSTINF-13	573	0	107	80	144	N/A
POSTINF-14	402	80	58.5	10	38.4	N/A
POSTINF-15	777	10	315	160	140	N/A
POSTINF-16	1030	160	293	160	213	N/A
POSTINF-17	1280	640	1210	80	651	N/A
POSTINF-18	140	10	69.2	320	82.1	N/A
POSTINF-19	101	20	41.3	10	24.9	N/A
POSTINF-20	234	20	116	10	78.1	N/A
POSTINF-21	651	40	147	40	31.6	160
POSTINF-22	269	40	54.2	40	22.9	40
POSTINF-23	1260	80	880	80	803	N/A
POSTINF-24	22.3	0	23.5	0	19.8	N/A
POSTINF-25	958	80	103	160	61.1	N/A
POSTINF-26	410	80	65.4	20	36.3	320
POSTINF-27	2950	160	833	160	638	N/A
POSTINF-28	82	160	15.7	0	10.4	160
POSTINF-29	2960	320	741	320	333	320
POSTINF-30	458	40	83.4	20	73.6	N/A
POSTINF-31	2040	320	613	160	639	N/A
POSTINF-32	1160	20	260	160	325	160
POSTINF-33	1490	10	231	80	130	N/A
POSTINF-34	1100	160	1030	160	458	N/A
POSTINF-35	4060	80	617	160	564	160
POSTINF-36	187	40	17.1	20	9.76	N/A
POSTINF-37	191	40	426	40	1270	80
POSTINF-38	314	10	82.7	10	24000	640
POSTINF-39	1360	80	317	160	13800	1280
POSTINF-40	220	40	59.7	20	2040	640

Supplementary Table S4. S-IgG concentrations of 43 subjects 0-16 weeks after vaccination.

ID	S-IgG concentration in BAU/ml over time (in weeks)							
	**0**	**1**	**2**	**3**	**4**	**5**	**8**	**16**
Homologous vaccination								
HOMVAC-1	4.96	80.1	1060	1110	6640	4140	2850	1830
HOMVAC-2	<4.81	<4.81	330	408	9870	10800	5040	1640
HOMVAC-3	<4.81	<4.81	89.8	64.8	1140	957	963	610
HOMVAC-4	<4.81	69.7	163	108	1400	1280	1090	705
HOMVAC-5	<4.81	<4.81	476	437	10500	9690	5880	1590
HOMVAC-6	<4.81	6.66	N/A	261	3990	N/A	2020	1220
HOMVAC-7	<4.81	<4.81	82.1	85	2130	2670	1780	397
HOMVAC-8	<4.81	12.5	1190	2660	12000	32700	14200	1770
HOMVAC-9	<4.81	N/A	45.8	51.9	1120	974	774	402
HOMVAC-10	<4.81	13.6	248	259	1910	1820	1280	489
HOMVAC-11	<4.81	9.84	159	106	1910	1650	742	250
HOMVAC-12	<4.81	<4.81	484	539	4930	3450	1580	891
HOMVAC-13	<4.81	<4.81	128	182	5310	4280	1640	1000
HOMVAC-14	<4.81	<4.81	200	103	1320	1310	710	249
HOMVAC-15	<4.81	<4.81	103	76.8	1000	762	486	270
HOMVAC-16	<4.81	35.1	263	228	6130	3980	2070	1230
HOMVAC-17	<4.81	<4.81	446	492	6720	6690	4240	1810
HOMVAC-18	<4.81	<4.81	391	446	4570	5360	N/A	1510
HOMVAC-19	<4.81	<4.81	317	298	2550	2290	1350	568
HOMVAC-20	N/A	<4.81	321	244	5350	3680	1730	1230
HOMVAC-21	N/A	<4.81	105	139	3750	4210	1900	1190
HOMVAC-22	<4.81	<4.81	148	136	1020	1970	1160	602
Heterologous vaccination								
HETVAC-1	<4.81	<4.81	66.8	122	150	248	357	4590
HETVAC-2	<4.81	<4.81	30.7	391	309	236	56.2	12600
HETVAC-3	<4.81	<4.81	66.3	193	177	N/A	111	3840
HETVAC-4	<4.81	<4.81	112	344	304	227	142	N/A
HETVAC-5	<4.81	<4.81	10.6	13.8	12.1	9.4	7.82	1500
HETVAC-6	8.59	8.81	68.1	170	N/A	138	95.5	1860
HETVAC-7	<4.81	<4.81	91.9	N/A	130	93.2	42.2	3000
HETVAC-8	<4.81	<4.81	8.41	26.2	37.1	43.4	41.5	2150
HETVAC-9	<4.81	<4.81	23.4	54	46.9	38.5	17.5	399
HETVAC-10	<4.81	<4.81	112	130	109	82.8	15.2	3150
HETVAC-11	<4.81	<4.81	21.1	83.6	N/A	86.9	30.5	5840
HETVAC-12	<4.81	<4.81	14.8	93.9	108	89.2	11.4	1030
HETVAC-13	19.2	1000	1770	1420	1140	1030	363	4150
HETVAC-14	<4.81	<4.81	215	N/A	273	221	45.2	3100
HETVAC-15	<4.81	<4.81	63.2	63.5	55.2	48.6	27.9	969
HETVAC-16	<4.81	<4.81	11	31.2	28.1	24.7	14.9	1960
HETVAC-17	<4.81	<4.81	152	381	313	260	129	9590
HETVAC-18	<4.81	<4.81	77.2	N/A	N/A	N/A	33.1	1460
HETVAC-19	<4.81	<4.81	28	56.6	64.3	54.7	21.4	1300
HETVAC-20	<4.81	<4.81	18.1	21.4	24.6	22.3	14.2	1390
HETVAC-21	<4.81	<4.81	206	336	313	283	123	5140
