# Supplementary figures and images for: Robust Neutralizing Antibody Levels Detected after Either SARS-CoV-2 Vaccination or One Year after Infection
Source: Viruses. 2021 Oct 5;13(10):2003. doi: 10.3390/v13102003 (PMC8537517; doi:10.3390/v13102003)

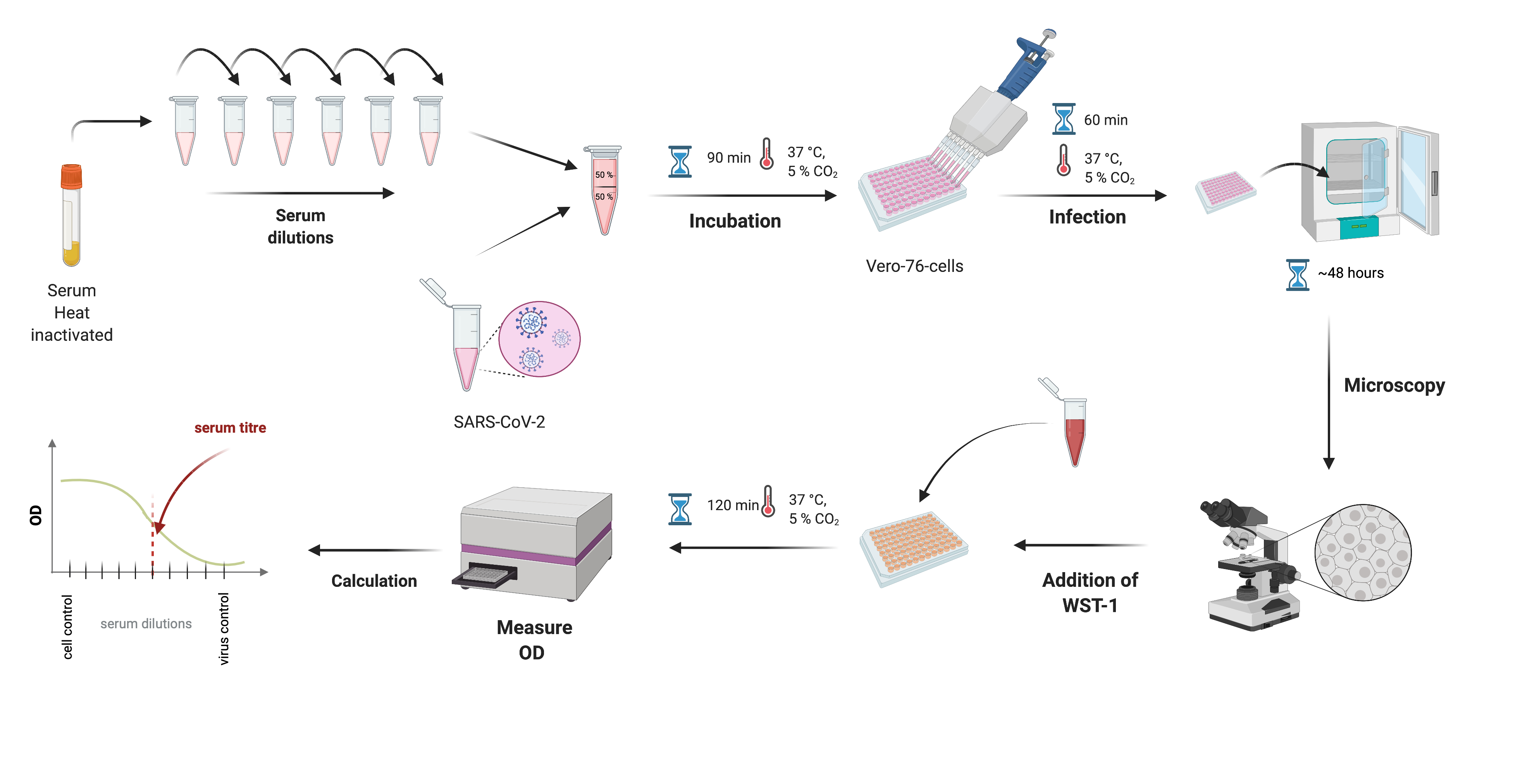

Supplement: Supplementary file 1 [file viruses-13-02003-s001.zip › Supplementary Figure S1.jpg]

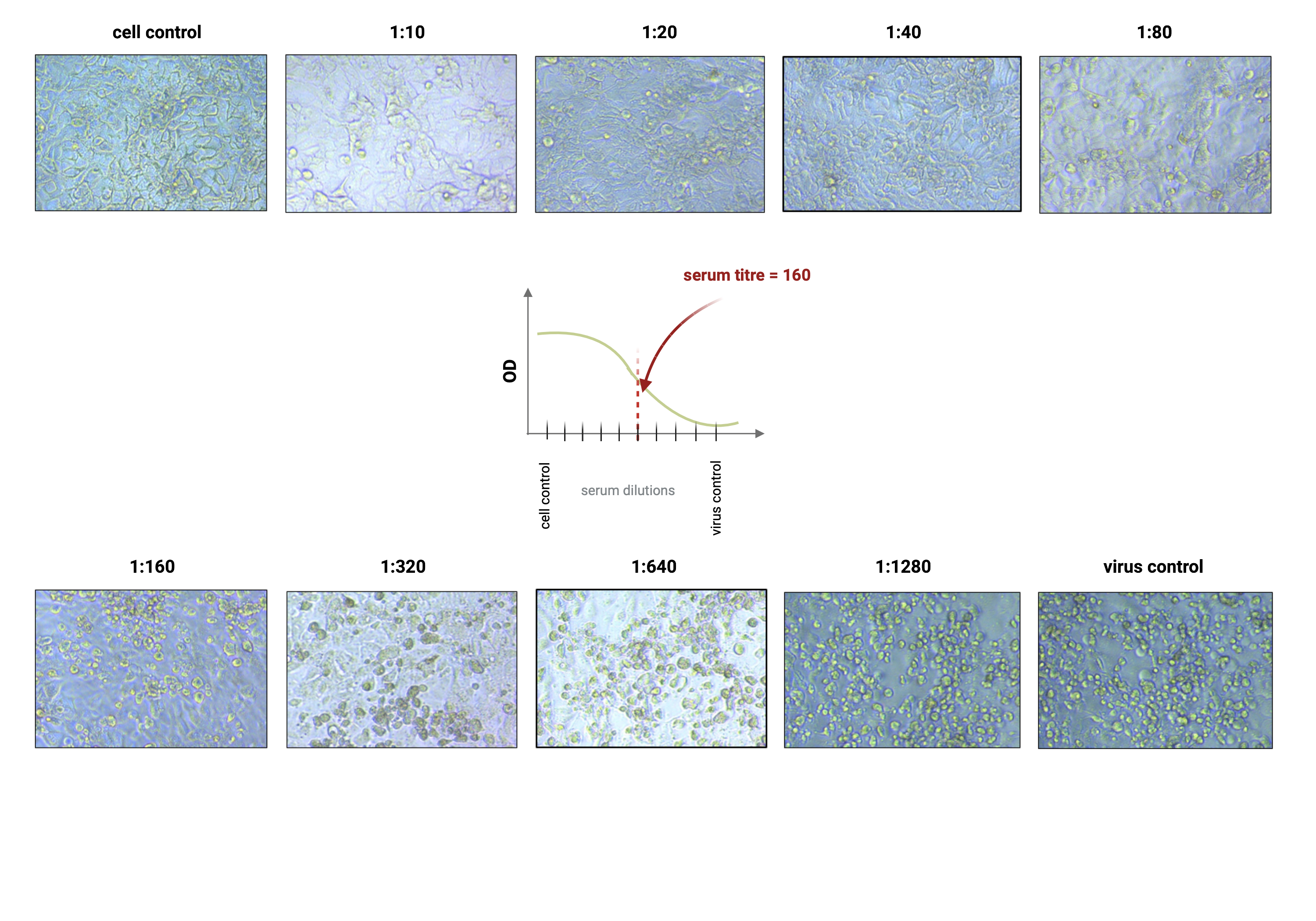

Supplement: Supplementary file 1 [file viruses-13-02003-s001.zip › Supplementary Figure S2.jpg]
